# Supplementary material for: Characterization of Amaranthus species: ability in nanoparticles fabrication and the antimicrobial activity against human pathogenic bacteria
Source: PeerJ. 2024 Feb 8;12:e16708. doi: 10.7717/peerj.16708 (PMC11075808; doi:10.7717/peerj.16708)
Supplement: Supplemental Information 1 [file peerj-12-16708-s001.docx]

**Table S1: Table presenting the antibacterial activity of AgNPs prepared by the different plant species. Data are mean ± SD of the inhibition zone (mm). Tukey's multiple comparisons test was used for comparison where different letter indicates significant variation.**

| **Bacterial species** | ***AgNPs- A. blitum subsp. blitum var. blitum (a)*** | ***AgNPs- A. graecizans L. subsp. Graecizans (b)*** | ***AgNPs-A. dubius (c)*** | ***AgNPs- A. blitoides var. blitoides (d)*** | ***AgNPS- A. viridis (e)*** | **Silver nitrate** | **Ampicillin** |
| --- | --- | --- | --- | --- | --- | --- | --- |
| *S. aureus* | 16±1^c^ | 18±1^c^ | 27±1^a^ | 24±1^b^ | 26±1^ab^ | 10.3±0.5^d^ | 12±0^d^ |
| *k. pneumoniae* | 18±1^b^ | 20±1^b^ | 19.7±1^b^ | 25±1^a^ | 20.3±0.5^c^ | 8.7±0.5^e^ | 13±0 ^d^ |
| *P. aeruginosa* | 17.3±1.5^b^ | 22.3±0.5^b^ | 26±1^a^ | 25±1^a^ | 27±1^a^ | 8.3±0.5^d^ | 11±0 ^c^ |
| *E. coli* | 20±1^c^ | 20±1^c^ | 26±1 ^b^ | 37±1 ^a^ | 28±1 ^b^ | 8.3±0.5 ^e^ | 13±0 ^d^ |

The effect of AgNPs prepared by different plant species, silver nitrate and ampicillin on different bacterial species was analyzed using Two-way ANOVA and the supporting analysis tables are given below (Supporting tables S2, S3 and S4).

Regarding the effect of different treatments against ***S. aureus***, differences were detected using two-way ANOVA. **Tukey's multiple comparisons test** revealed that significant differences existed among all tested agents except between AgNPs prepared by *A. dubius* and *A. viridis (*c and e*); A. blitoides and blitoides vs. A. viridis (*d and e*); A. blitum subsp. blitum and blitum vs A. graecizans L. subsp. Graecizans (*a and b*)* and Silver nitrate and Ampicillin*.* However, after performing the LSD test, three of this group were shown to be significantly different (*A. blitoides* and *blitoides vs. A. viridis (*d and e*); A. blitum subsp. blitum and blitum vs A. graecizans L. subsp. Graecizans (*a and b*)* and silver nitrate and Ampicillin.

**For *k. pneumoniae,* Tukey's multiple comparisons test indicated** significant differences existed among all tested agents except between *A. blitum subsp. blitum var. blitum and A. graecizans L. subsp. Graecizans (a and b); A. blitum subsp. blitum var. blitum and A. dubius (*a and c*); A. graecizans L. subsp. Graecizans and. A. dubius (*b and c*); A. graecizans L. subsp. Graecizans* and*. A. viridis (b and e)* and *A. dubius vs. A. viridis (*c and d*).* However, after performing the LSD, tow of this group were shown to be significantly different (*A. blitum subsp. blitum var. blitum and A. graecizans L. subsp. Graecizans (a and b) A. blitum subsp. blitum var. blitum and A. dubius (*a and c).

**For *P. aeruginosa,* Tukey's multiple comparisons test indicated** significant differences existed among all tested agents except between *A. dubius* and *A. blitoides var. blitoides (*c and d*); A. dubius* and *A. viridis* (c and e) and *A. blitoides var. blitoides and. A. viridis* (d and e)*.* However, comparison between *A. blitoides var. blitoides and. A. viridis* (d and e) showed significant variation after performing the LSD test.

**For *E. coli,* Tukey's multiple comparisons test indicated** significant differences existed among all tested agents except between *A. blitum subsp. blitum var. blitum* and *A. graecizans L. subsp. Graecizans (a and b) and between A. dubius and. A. viridis (c and e)* however*,* When LSD analysis was performed *A. dubius and. A. viridis (c and e)* comparison was significant.

**Table S2: The ANOVA table**

|  |  |  |  |  |  |
| --- | --- | --- | --- | --- | --- |
|  |  |  |  |  |  |
| Data summary |  |  |  |  |  |
| Number of columns (Column Factor) | 7 |  |  |  |  |
| Number of rows (Row Factor) | 4 |  |  |  |  |
| Number of values | 84 |  |  |  |  |
|  |  |  |  |  |  |
| Table Analyzed | Data 1 |  |  |  |  |
|  |  |  |  |  |  |
| Two-way ANOVA | Ordinary |  |  |  |  |
| Alpha | 0.05 |  |  |  |  |
|  |  |  |  |  |  |
| Source of Variation | % of total variation | P value | P value summary | Significant? |  |
| Interaction | 10.67 | <0.0001 | **** | Yes |  |
| Row Factor | 4.067 | <0.0001 | **** | Yes |  |
| Column Factor | 84.28 | <0.0001 | **** | Yes |  |
|  |  |  |  |  |  |
| ANOVA table | SS | DF | MS | F (DFn, DFd) | P value |
| Interaction | 450.3 | 18 | 25.02 | F (18, 56) = 33.90 | P<0.0001 |
| Row Factor | 171.7 | 3 | 57.22 | F (3, 56) = 77.53 | P<0.0001 |
| Column Factor | 3557 | 6 | 592.9 | F (6, 56) = 803.3 | P<0.0001 |
| Residual | 41.33 | 56 | 0.7381 |  |  |
|  |  |  |  |  |  |

**Table S3: Two-way ANOVA, Tukey's multiple comparisons test for comparing the effect of the tested agents against *S. aureus, k. pneumoniae, P. aeruginosa* and *E. coil*.**

|  |  |  |  |  |  |  |  |  |
| --- | --- | --- | --- | --- | --- | --- | --- | --- |
| TWO-WAY ANOVA Within each row, compare columns (simple effects within rows) |  |  |  |  |  |  |  |  |
|  |  |  |  |  |  |  |  |  |
| Number of families | 4 |  |  |  |  |  |  |  |
| Number of comparisons per family | 21 |  |  |  |  |  |  |  |
| Alpha | 0.05 |  |  |  |  |  |  |  |
|  |  |  |  |  |  |  |  |  |
| Tukey's multiple comparisons test | Mean Diff. | 95.00% CI of diff. | Below threshold? | Summary | Adjusted P Value |  |  |  |
|  |  |  |  |  |  |  |  |  |
| S. aureus |  |  |  |  |  |  |  |  |
| Group A vs. Group B | -2.000 | -4.145 to 0.1451 | No | ns | 0.0830 |  |  |  |
| Group A vs. A. dubius | -11.00 | -13.15 to -8.855 | Yes | **** | <0.0001 |  |  |  |
| Group A vs. A. blitoides var. blitoides | -8.000 | -10.15 to -5.855 | Yes | **** | <0.0001 |  |  |  |
| Group A vs. A. viridis | -10.00 | -12.15 to -7.855 | Yes | **** | <0.0001 |  |  |  |
| Group A vs. Silver nitrate | 5.667 | 3.522 to 7.812 | Yes | **** | <0.0001 |  |  |  |
| Group A vs. Ampicillin | 4.000 | 1.855 to 6.145 | Yes | **** | <0.0001 |  |  |  |
| Group B vs. A. dubius | -9.000 | -11.15 to -6.855 | Yes | **** | <0.0001 |  |  |  |
| Group B vs. A. blitoides var. blitoides | -6.000 | -8.145 to -3.855 | Yes | **** | <0.0001 |  |  |  |
| Group B vs. A. viridis | -8.000 | -10.15 to -5.855 | Yes | **** | <0.0001 |  |  |  |
| Group B vs. Silver nitrate | 7.667 | 5.522 to 9.812 | Yes | **** | <0.0001 |  |  |  |
| Group B vs. Ampicillin | 6.000 | 3.855 to 8.145 | Yes | **** | <0.0001 |  |  |  |
| A. dubius vs. A. blitoides var. blitoides | 3.000 | 0.8549 to 5.145 | Yes | ** | 0.0014 |  |  |  |
| A. dubius vs. A. viridis | 1.000 | -1.145 to 3.145 | No | ns | 0.7858 |  |  |  |
| A. dubius vs. Silver nitrate | 16.67 | 14.52 to 18.81 | Yes | **** | <0.0001 |  |  |  |
| A. dubius vs. Ampicillin | 15.00 | 12.85 to 17.15 | Yes | **** | <0.0001 |  |  |  |
| A. blitoides var. blitoides vs. A. viridis | -2.000 | -4.145 to 0.1451 | No | ns | 0.0830 |  |  |  |
| A. blitoides var. blitoides vs. Silver nitrate | 13.67 | 11.52 to 15.81 | Yes | **** | <0.0001 |  |  |  |
| A. blitoides var. blitoides vs. Ampicillin | 12.00 | 9.855 to 14.15 | Yes | **** | <0.0001 |  |  |  |
| A. viridis vs. Silver nitrate | 15.67 | 13.52 to 17.81 | Yes | **** | <0.0001 |  |  |  |
| A. viridis vs. Ampicillin | 14.00 | 11.85 to 16.15 | Yes | **** | <0.0001 |  |  |  |
| Silver nitrate vs. Ampicillin | -1.667 | -3.812 to 0.4784 | No | ns | 0.2281 |  |  |  |
|  |  |  |  |  |  |  |  |  |
| k. pneumoniae |  |  |  |  |  |  |  |  |
| Group A vs. Group B | -2.000 | -4.145 to 0.1451 | No | ns | 0.0830 |  |  |  |
| Group A vs. A. dubius | -1.667 | -3.812 to 0.4784 | No | ns | 0.2281 |  |  |  |
| Group A vs. A. blitoides var. blitoides | -7.000 | -9.145 to -4.855 | Yes | **** | <0.0001 |  |  |  |
| Group A vs. A. viridis | -2.333 | -4.478 to -0.1882 | Yes | * | 0.0246 |  |  |  |
| Group A vs. Silver nitrate | 9.333 | 7.188 to 11.48 | Yes | **** | <0.0001 |  |  |  |
| Group A vs. Ampicillin | 5.000 | 2.855 to 7.145 | Yes | **** | <0.0001 |  |  |  |
| Group B vs. A. dubius | 0.3333 | -1.812 to 2.478 | No | ns | 0.9991 |  |  |  |
| Group B vs. A. blitoides var. blitoides | -5.000 | -7.145 to -2.855 | Yes | **** | <0.0001 |  |  |  |
| Group B vs. A. viridis | -0.3333 | -2.478 to 1.812 | No | ns | 0.9991 |  |  |  |
| Group B vs. Silver nitrate | 11.33 | 9.188 to 13.48 | Yes | **** | <0.0001 |  |  |  |
| Group B vs. Ampicillin | 7.000 | 4.855 to 9.145 | Yes | **** | <0.0001 |  |  |  |
| A. dubius vs. A. blitoides var. blitoides | -5.333 | -7.478 to -3.188 | Yes | **** | <0.0001 |  |  |  |
| A. dubius vs. A. viridis | -0.6667 | -2.812 to 1.478 | No | ns | 0.9623 |  |  |  |
| A. dubius vs. Silver nitrate | 11.00 | 8.855 to 13.15 | Yes | **** | <0.0001 |  |  |  |
| A. dubius vs. Ampicillin | 6.667 | 4.522 to 8.812 | Yes | **** | <0.0001 |  |  |  |
| A. blitoides var. blitoides vs. A. viridis | 4.667 | 2.522 to 6.812 | Yes | **** | <0.0001 |  |  |  |
| A. blitoides var. blitoides vs. Silver nitrate | 16.33 | 14.19 to 18.48 | Yes | **** | <0.0001 |  |  |  |
| A. blitoides var. blitoides vs. Ampicillin | 12.00 | 9.855 to 14.15 | Yes | **** | <0.0001 |  |  |  |
| A. viridis vs. Silver nitrate | 11.67 | 9.522 to 13.81 | Yes | **** | <0.0001 |  |  |  |
| A. viridis vs. Ampicillin | 7.333 | 5.188 to 9.478 | Yes | **** | <0.0001 |  |  |  |
| Silver nitrate vs. Ampicillin | -4.333 | -6.478 to -2.188 | Yes | **** | <0.0001 |  |  |  |
|  |  |  |  |  |  |  |  |  |
| P. aeruginosa |  |  |  |  |  |  |  |  |
| Group A vs. Group B | -5.000 | -7.145 to -2.855 | Yes | **** | <0.0001 |  |  |  |
| Group A vs. A. dubius | -8.667 | -10.81 to -6.522 | Yes | **** | <0.0001 |  |  |  |
| Group A vs. A. blitoides var. blitoides | -7.667 | -9.812 to -5.522 | Yes | **** | <0.0001 |  |  |  |
| Group A vs. A. viridis | -9.667 | -11.81 to -7.522 | Yes | **** | <0.0001 |  |  |  |
| Group A vs. Silver nitrate | 9.000 | 6.855 to 11.15 | Yes | **** | <0.0001 |  |  |  |
| Group A vs. Ampicillin | 6.333 | 4.188 to 8.478 | Yes | **** | <0.0001 |  |  |  |
| Group B vs. A. dubius | -3.667 | -5.812 to -1.522 | Yes | **** | <0.0001 |  |  |  |
| Group B vs. A. blitoides var. blitoides | -2.667 | -4.812 to -0.5216 | Yes | ** | 0.0062 |  |  |  |
| Group B vs. A. viridis | -4.667 | -6.812 to -2.522 | Yes | **** | <0.0001 |  |  |  |
| Group B vs. Silver nitrate | 14.00 | 11.85 to 16.15 | Yes | **** | <0.0001 |  |  |  |
| Group B vs. Ampicillin | 11.33 | 9.188 to 13.48 | Yes | **** | <0.0001 |  |  |  |
| A. dubius vs. A. blitoides var. blitoides | 1.000 | -1.145 to 3.145 | No | ns | 0.7858 |  |  |  |
| A. dubius vs. A. viridis | -1.000 | -3.145 to 1.145 | No | ns | 0.7858 |  |  |  |
| A. dubius vs. Silver nitrate | 17.67 | 15.52 to 19.81 | Yes | **** | <0.0001 |  |  |  |
| A. dubius vs. Ampicillin | 15.00 | 12.85 to 17.15 | Yes | **** | <0.0001 |  |  |  |
| A. blitoides var. blitoides vs. A. viridis | -2.000 | -4.145 to 0.1451 | No | ns | 0.0830 |  |  |  |
| A. blitoides var. blitoides vs. Silver nitrate | 16.67 | 14.52 to 18.81 | Yes | **** | <0.0001 |  |  |  |
| A. blitoides var. blitoides vs. Ampicillin | 14.00 | 11.85 to 16.15 | Yes | **** | <0.0001 |  |  |  |
| A. viridis vs. Silver nitrate | 18.67 | 16.52 to 20.81 | Yes | **** | <0.0001 |  |  |  |
| A. viridis vs. Ampicillin | 16.00 | 13.85 to 18.15 | Yes | **** | <0.0001 |  |  |  |
| Silver nitrate vs. Ampicillin | -2.667 | -4.812 to -0.5216 | Yes | ** | 0.0062 |  |  |  |
|  |  |  |  |  |  |  |  |  |
| E. coli |  |  |  |  |  |  |  |  |
| Group A vs. Group B | 0.000 | -2.145 to 2.145 | No | ns | >0.9999 |  |  |  |
| Group A vs. A. dubius | -6.000 | -8.145 to -3.855 | Yes | **** | <0.0001 |  |  |  |
| Group A vs. A. blitoides var. blitoides | -17.00 | -19.15 to -14.85 | Yes | **** | <0.0001 |  |  |  |
| Group A vs. A. viridis | -8.000 | -10.15 to -5.855 | Yes | **** | <0.0001 |  |  |  |
| Group A vs. Silver nitrate | 11.67 | 9.522 to 13.81 | Yes | **** | <0.0001 |  |  |  |
| Group A vs. Ampicillin | 7.000 | 4.855 to 9.145 | Yes | **** | <0.0001 |  |  |  |
| Group B vs. A. dubius | -6.000 | -8.145 to -3.855 | Yes | **** | <0.0001 |  |  |  |
| Group B vs. A. blitoides var. blitoides | -17.00 | -19.15 to -14.85 | Yes | **** | <0.0001 |  |  |  |
| Group B vs. A. viridis | -8.000 | -10.15 to -5.855 | Yes | **** | <0.0001 |  |  |  |
| Group B vs. Silver nitrate | 11.67 | 9.522 to 13.81 | Yes | **** | <0.0001 |  |  |  |
| Group B vs. Ampicillin | 7.000 | 4.855 to 9.145 | Yes | **** | <0.0001 |  |  |  |
| A. dubius vs. A. blitoides var. blitoides | -11.00 | -13.15 to -8.855 | Yes | **** | <0.0001 |  |  |  |
| A. dubius vs. A. viridis | -2.000 | -4.145 to 0.1451 | No | ns | 0.0830 |  |  |  |
| A. dubius vs. Silver nitrate | 17.67 | 15.52 to 19.81 | Yes | **** | <0.0001 |  |  |  |
| A. dubius vs. Ampicillin | 13.00 | 10.85 to 15.15 | Yes | **** | <0.0001 |  |  |  |
| A. blitoides var. blitoides vs. A. viridis | 9.000 | 6.855 to 11.15 | Yes | **** | <0.0001 |  |  |  |
| A. blitoides var. blitoides vs. Silver nitrate | 28.67 | 26.52 to 30.81 | Yes | **** | <0.0001 |  |  |  |
| A. blitoides var. blitoides vs. Ampicillin | 24.00 | 21.85 to 26.15 | Yes | **** | <0.0001 |  |  |  |
| A. viridis vs. Silver nitrate | 19.67 | 17.52 to 21.81 | Yes | **** | <0.0001 |  |  |  |
| A. viridis vs. Ampicillin | 15.00 | 12.85 to 17.15 | Yes | **** | <0.0001 |  |  |  |
| Silver nitrate vs. Ampicillin | -4.667 | -6.812 to -2.522 | Yes | **** | <0.0001 |  |  |  |
|  |  |  |  |  |  |  |  |  |
|  |  |  |  |  |  |  |  |  |
| Test details | Mean 1 | Mean 2 | Mean Diff. | SE of diff. | N1 | N2 | q | DF |
|  |  |  |  |  |  |  |  |  |
| S. aureus |  |  |  |  |  |  |  |  |
| Group A vs. Group B | 16.00 | 18.00 | -2.000 | 0.7015 | 3 | 3 | 4.032 | 56.00 |
| Group A vs. A. dubius | 16.00 | 27.00 | -11.00 | 0.7015 | 3 | 3 | 22.18 | 56.00 |
| Group A vs. A. blitoides var. blitoides | 16.00 | 24.00 | -8.000 | 0.7015 | 3 | 3 | 16.13 | 56.00 |
| Group A vs. A. viridis | 16.00 | 26.00 | -10.00 | 0.7015 | 3 | 3 | 20.16 | 56.00 |
| Group A vs. Silver nitrate | 16.00 | 10.33 | 5.667 | 0.7015 | 3 | 3 | 11.42 | 56.00 |
| Group A vs. Ampicillin | 16.00 | 12.00 | 4.000 | 0.7015 | 3 | 3 | 8.064 | 56.00 |
| Group B vs. A. dubius | 18.00 | 27.00 | -9.000 | 0.7015 | 3 | 3 | 18.14 | 56.00 |
| Group B vs. A. blitoides var. blitoides | 18.00 | 24.00 | -6.000 | 0.7015 | 3 | 3 | 12.10 | 56.00 |
| Group B vs. A. viridis | 18.00 | 26.00 | -8.000 | 0.7015 | 3 | 3 | 16.13 | 56.00 |
| Group B vs. Silver nitrate | 18.00 | 10.33 | 7.667 | 0.7015 | 3 | 3 | 15.46 | 56.00 |
| Group B vs. Ampicillin | 18.00 | 12.00 | 6.000 | 0.7015 | 3 | 3 | 12.10 | 56.00 |
| A. dubius vs. A. blitoides var. blitoides | 27.00 | 24.00 | 3.000 | 0.7015 | 3 | 3 | 6.048 | 56.00 |
| A. dubius vs. A. viridis | 27.00 | 26.00 | 1.000 | 0.7015 | 3 | 3 | 2.016 | 56.00 |
| A. dubius vs. Silver nitrate | 27.00 | 10.33 | 16.67 | 0.7015 | 3 | 3 | 33.60 | 56.00 |
| A. dubius vs. Ampicillin | 27.00 | 12.00 | 15.00 | 0.7015 | 3 | 3 | 30.24 | 56.00 |
| A. blitoides var. blitoides vs. A. viridis | 24.00 | 26.00 | -2.000 | 0.7015 | 3 | 3 | 4.032 | 56.00 |
| A. blitoides var. blitoides vs. Silver nitrate | 24.00 | 10.33 | 13.67 | 0.7015 | 3 | 3 | 27.55 | 56.00 |
| A. blitoides var. blitoides vs. Ampicillin | 24.00 | 12.00 | 12.00 | 0.7015 | 3 | 3 | 24.19 | 56.00 |
| A. viridis vs. Silver nitrate | 26.00 | 10.33 | 15.67 | 0.7015 | 3 | 3 | 31.59 | 56.00 |
| A. viridis vs. Ampicillin | 26.00 | 12.00 | 14.00 | 0.7015 | 3 | 3 | 28.22 | 56.00 |
| Silver nitrate vs. Ampicillin | 10.33 | 12.00 | -1.667 | 0.7015 | 3 | 3 | 3.360 | 56.00 |
|  |  |  |  |  |  |  |  |  |
| k. pneumoniae |  |  |  |  |  |  |  |  |
| Group A vs. Group B | 18.00 | 20.00 | -2.000 | 0.7015 | 3 | 3 | 4.032 | 56.00 |
| Group A vs. A. dubius | 18.00 | 19.67 | -1.667 | 0.7015 | 3 | 3 | 3.360 | 56.00 |
| Group A vs. A. blitoides var. blitoides | 18.00 | 25.00 | -7.000 | 0.7015 | 3 | 3 | 14.11 | 56.00 |
| Group A vs. A. viridis | 18.00 | 20.33 | -2.333 | 0.7015 | 3 | 3 | 4.704 | 56.00 |
| Group A vs. Silver nitrate | 18.00 | 8.667 | 9.333 | 0.7015 | 3 | 3 | 18.82 | 56.00 |
| Group A vs. Ampicillin | 18.00 | 13.00 | 5.000 | 0.7015 | 3 | 3 | 10.08 | 56.00 |
| Group B vs. A. dubius | 20.00 | 19.67 | 0.3333 | 0.7015 | 3 | 3 | 0.6720 | 56.00 |
| Group B vs. A. blitoides var. blitoides | 20.00 | 25.00 | -5.000 | 0.7015 | 3 | 3 | 10.08 | 56.00 |
| Group B vs. A. viridis | 20.00 | 20.33 | -0.3333 | 0.7015 | 3 | 3 | 0.6720 | 56.00 |
| Group B vs. Silver nitrate | 20.00 | 8.667 | 11.33 | 0.7015 | 3 | 3 | 22.85 | 56.00 |
| Group B vs. Ampicillin | 20.00 | 13.00 | 7.000 | 0.7015 | 3 | 3 | 14.11 | 56.00 |
| A. dubius vs. A. blitoides var. blitoides | 19.67 | 25.00 | -5.333 | 0.7015 | 3 | 3 | 10.75 | 56.00 |
| A. dubius vs. A. viridis | 19.67 | 20.33 | -0.6667 | 0.7015 | 3 | 3 | 1.344 | 56.00 |
| A. dubius vs. Silver nitrate | 19.67 | 8.667 | 11.00 | 0.7015 | 3 | 3 | 22.18 | 56.00 |
| A. dubius vs. Ampicillin | 19.67 | 13.00 | 6.667 | 0.7015 | 3 | 3 | 13.44 | 56.00 |
| A. blitoides var. blitoides vs. A. viridis | 25.00 | 20.33 | 4.667 | 0.7015 | 3 | 3 | 9.408 | 56.00 |
| A. blitoides var. blitoides vs. Silver nitrate | 25.00 | 8.667 | 16.33 | 0.7015 | 3 | 3 | 32.93 | 56.00 |
| A. blitoides var. blitoides vs. Ampicillin | 25.00 | 13.00 | 12.00 | 0.7015 | 3 | 3 | 24.19 | 56.00 |
| A. viridis vs. Silver nitrate | 20.33 | 8.667 | 11.67 | 0.7015 | 3 | 3 | 23.52 | 56.00 |
| A. viridis vs. Ampicillin | 20.33 | 13.00 | 7.333 | 0.7015 | 3 | 3 | 14.78 | 56.00 |
| Silver nitrate vs. Ampicillin | 8.667 | 13.00 | -4.333 | 0.7015 | 3 | 3 | 8.736 | 56.00 |
|  |  |  |  |  |  |  |  |  |
| P. aeruginosa |  |  |  |  |  |  |  |  |
| Group A vs. Group B | 17.33 | 22.33 | -5.000 | 0.7015 | 3 | 3 | 10.08 | 56.00 |
| Group A vs. A. dubius | 17.33 | 26.00 | -8.667 | 0.7015 | 3 | 3 | 17.47 | 56.00 |
| Group A vs. A. blitoides var. blitoides | 17.33 | 25.00 | -7.667 | 0.7015 | 3 | 3 | 15.46 | 56.00 |
| Group A vs. A. viridis | 17.33 | 27.00 | -9.667 | 0.7015 | 3 | 3 | 19.49 | 56.00 |
| Group A vs. Silver nitrate | 17.33 | 8.333 | 9.000 | 0.7015 | 3 | 3 | 18.14 | 56.00 |
| Group A vs. Ampicillin | 17.33 | 11.00 | 6.333 | 0.7015 | 3 | 3 | 12.77 | 56.00 |
| Group B vs. A. dubius | 22.33 | 26.00 | -3.667 | 0.7015 | 3 | 3 | 7.392 | 56.00 |
| Group B vs. A. blitoides var. blitoides | 22.33 | 25.00 | -2.667 | 0.7015 | 3 | 3 | 5.376 | 56.00 |
| Group B vs. A. viridis | 22.33 | 27.00 | -4.667 | 0.7015 | 3 | 3 | 9.408 | 56.00 |
| Group B vs. Silver nitrate | 22.33 | 8.333 | 14.00 | 0.7015 | 3 | 3 | 28.22 | 56.00 |
| Group B vs. Ampicillin | 22.33 | 11.00 | 11.33 | 0.7015 | 3 | 3 | 22.85 | 56.00 |
| A. dubius vs. A. blitoides var. blitoides | 26.00 | 25.00 | 1.000 | 0.7015 | 3 | 3 | 2.016 | 56.00 |
| A. dubius vs. A. viridis | 26.00 | 27.00 | -1.000 | 0.7015 | 3 | 3 | 2.016 | 56.00 |
| A. dubius vs. Silver nitrate | 26.00 | 8.333 | 17.67 | 0.7015 | 3 | 3 | 35.62 | 56.00 |
| A. dubius vs. Ampicillin | 26.00 | 11.00 | 15.00 | 0.7015 | 3 | 3 | 30.24 | 56.00 |
| A. blitoides var. blitoides vs. A. viridis | 25.00 | 27.00 | -2.000 | 0.7015 | 3 | 3 | 4.032 | 56.00 |
| A. blitoides var. blitoides vs. Silver nitrate | 25.00 | 8.333 | 16.67 | 0.7015 | 3 | 3 | 33.60 | 56.00 |
| A. blitoides var. blitoides vs. Ampicillin | 25.00 | 11.00 | 14.00 | 0.7015 | 3 | 3 | 28.22 | 56.00 |
| A. viridis vs. Silver nitrate | 27.00 | 8.333 | 18.67 | 0.7015 | 3 | 3 | 37.63 | 56.00 |
| A. viridis vs. Ampicillin | 27.00 | 11.00 | 16.00 | 0.7015 | 3 | 3 | 32.26 | 56.00 |
| Silver nitrate vs. Ampicillin | 8.333 | 11.00 | -2.667 | 0.7015 | 3 | 3 | 5.376 | 56.00 |
|  |  |  |  |  |  |  |  |  |
| E. coli |  |  |  |  |  |  |  |  |
| Group A vs. Group B | 20.00 | 20.00 | 0.000 | 0.7015 | 3 | 3 | 0.000 | 56.00 |
| Group A vs. A. dubius | 20.00 | 26.00 | -6.000 | 0.7015 | 3 | 3 | 12.10 | 56.00 |
| Group A vs. A. blitoides var. blitoides | 20.00 | 37.00 | -17.00 | 0.7015 | 3 | 3 | 34.27 | 56.00 |
| Group A vs. A. viridis | 20.00 | 28.00 | -8.000 | 0.7015 | 3 | 3 | 16.13 | 56.00 |
| Group A vs. Silver nitrate | 20.00 | 8.333 | 11.67 | 0.7015 | 3 | 3 | 23.52 | 56.00 |
| Group A vs. Ampicillin | 20.00 | 13.00 | 7.000 | 0.7015 | 3 | 3 | 14.11 | 56.00 |
| Group B vs. A. dubius | 20.00 | 26.00 | -6.000 | 0.7015 | 3 | 3 | 12.10 | 56.00 |
| Group B vs. A. blitoides var. blitoides | 20.00 | 37.00 | -17.00 | 0.7015 | 3 | 3 | 34.27 | 56.00 |
| Group B vs. A. viridis | 20.00 | 28.00 | -8.000 | 0.7015 | 3 | 3 | 16.13 | 56.00 |
| Group B vs. Silver nitrate | 20.00 | 8.333 | 11.67 | 0.7015 | 3 | 3 | 23.52 | 56.00 |
| Group B vs. Ampicillin | 20.00 | 13.00 | 7.000 | 0.7015 | 3 | 3 | 14.11 | 56.00 |
| A. dubius vs. A. blitoides var. blitoides | 26.00 | 37.00 | -11.00 | 0.7015 | 3 | 3 | 22.18 | 56.00 |
| A. dubius vs. A. viridis | 26.00 | 28.00 | -2.000 | 0.7015 | 3 | 3 | 4.032 | 56.00 |
| A. dubius vs. Silver nitrate | 26.00 | 8.333 | 17.67 | 0.7015 | 3 | 3 | 35.62 | 56.00 |
| A. dubius vs. Ampicillin | 26.00 | 13.00 | 13.00 | 0.7015 | 3 | 3 | 26.21 | 56.00 |
| A. blitoides var. blitoides vs. A. viridis | 37.00 | 28.00 | 9.000 | 0.7015 | 3 | 3 | 18.14 | 56.00 |
| A. blitoides var. blitoides vs. Silver nitrate | 37.00 | 8.333 | 28.67 | 0.7015 | 3 | 3 | 57.79 | 56.00 |
| A. blitoides var. blitoides vs. Ampicillin | 37.00 | 13.00 | 24.00 | 0.7015 | 3 | 3 | 48.39 | 56.00 |
| A. viridis vs. Silver nitrate | 28.00 | 8.333 | 19.67 | 0.7015 | 3 | 3 | 39.65 | 56.00 |
| A. viridis vs. Ampicillin | 28.00 | 13.00 | 15.00 | 0.7015 | 3 | 3 | 30.24 | 56.00 |
| Silver nitrate vs. Ampicillin | 8.333 | 13.00 | -4.667 | 0.7015 | 3 | 3 | 9.408 | 56.00 |

**Table S4:** Fisher's LSD test for comparing the effect of the tested agents against *S. aureus, k. pneumoniae, P. aeruginosa* and *E. coil*.

| TWO-WAY ANOVA |  |  |  |  |  |  |  |  |
| --- | --- | --- | --- | --- | --- | --- | --- | --- |
| Within each row, compare columns (simple effects within rows) |  |  |  |  |  |  |  |  |
|  |  |  |  |  |  |  |  |  |
| Number of families | 4 |  |  |  |  |  |  |  |
| Number of comparisons per family | 21 |  |  |  |  |  |  |  |
| Alpha | 0.05 |  |  |  |  |  |  |  |
|  |  |  |  |  |  |  |  |  |
| Uncorrected Fisher's LSD | Mean Diff. | 95.00% CI of diff. | Below threshold? | Summary | Individual P Value |  |  |  |
|  |  |  |  |  |  |  |  |  |
| S. aureus |  |  |  |  |  |  |  |  |
| Group A vs. Group B | -2.000 | -3.405 to -0.5948 | Yes | ** | 0.0061 |  |  |  |
| Group A vs. A. dubius | -11.00 | -12.41 to -9.595 | Yes | **** | <0.0001 |  |  |  |
| Group A vs. A. blitoides var. blitoides | -8.000 | -9.405 to -6.595 | Yes | **** | <0.0001 |  |  |  |
| Group A vs. A. viridis | -10.00 | -11.41 to -8.595 | Yes | **** | <0.0001 |  |  |  |
| Group A vs. Silver nitrate | 5.667 | 4.261 to 7.072 | Yes | **** | <0.0001 |  |  |  |
| Group A vs. Ampicillin | 4.000 | 2.595 to 5.405 | Yes | **** | <0.0001 |  |  |  |
| Group B vs. A. dubius | -9.000 | -10.41 to -7.595 | Yes | **** | <0.0001 |  |  |  |
| Group B vs. A. blitoides var. blitoides | -6.000 | -7.405 to -4.595 | Yes | **** | <0.0001 |  |  |  |
| Group B vs. A. viridis | -8.000 | -9.405 to -6.595 | Yes | **** | <0.0001 |  |  |  |
| Group B vs. Silver nitrate | 7.667 | 6.261 to 9.072 | Yes | **** | <0.0001 |  |  |  |
| Group B vs. Ampicillin | 6.000 | 4.595 to 7.405 | Yes | **** | <0.0001 |  |  |  |
| A. dubius vs. A. blitoides var. blitoides | 3.000 | 1.595 to 4.405 | Yes | **** | <0.0001 |  |  |  |
| A. dubius vs. A. viridis | 1.000 | -0.4052 to 2.405 | No | ns | 0.1595 |  |  |  |
| A. dubius vs. Silver nitrate | 16.67 | 15.26 to 18.07 | Yes | **** | <0.0001 |  |  |  |
| A. dubius vs. Ampicillin | 15.00 | 13.59 to 16.41 | Yes | **** | <0.0001 |  |  |  |
| A. blitoides var. blitoides vs. A. viridis | -2.000 | -3.405 to -0.5948 | Yes | ** | 0.0061 |  |  |  |
| A. blitoides var. blitoides vs. Silver nitrate | 13.67 | 12.26 to 15.07 | Yes | **** | <0.0001 |  |  |  |
| A. blitoides var. blitoides vs. Ampicillin | 12.00 | 10.59 to 13.41 | Yes | **** | <0.0001 |  |  |  |
| A. viridis vs. Silver nitrate | 15.67 | 14.26 to 17.07 | Yes | **** | <0.0001 |  |  |  |
| A. viridis vs. Ampicillin | 14.00 | 12.59 to 15.41 | Yes | **** | <0.0001 |  |  |  |
| Silver nitrate vs. Ampicillin | -1.667 | -3.072 to -0.2614 | Yes | * | 0.0209 |  |  |  |
|  |  |  |  |  |  |  |  |  |
| k. pneumoniae |  |  |  |  |  |  |  |  |
| Group A vs. Group B | -2.000 | -3.405 to -0.5948 | Yes | ** | 0.0061 |  |  |  |
| Group A vs. A. dubius | -1.667 | -3.072 to -0.2614 | Yes | * | 0.0209 |  |  |  |
| Group A vs. A. blitoides var. blitoides | -7.000 | -8.405 to -5.595 | Yes | **** | <0.0001 |  |  |  |
| Group A vs. A. viridis | -2.333 | -3.739 to -0.9281 | Yes | ** | 0.0016 |  |  |  |
| Group A vs. Silver nitrate | 9.333 | 7.928 to 10.74 | Yes | **** | <0.0001 |  |  |  |
| Group A vs. Ampicillin | 5.000 | 3.595 to 6.405 | Yes | **** | <0.0001 |  |  |  |
| Group B vs. A. dubius | 0.3333 | -1.072 to 1.739 | No | ns | 0.6365 |  |  |  |
| Group B vs. A. blitoides var. blitoides | -5.000 | -6.405 to -3.595 | Yes | **** | <0.0001 |  |  |  |
| Group B vs. A. viridis | -0.3333 | -1.739 to 1.072 | No | ns | 0.6365 |  |  |  |
| Group B vs. Silver nitrate | 11.33 | 9.928 to 12.74 | Yes | **** | <0.0001 |  |  |  |
| Group B vs. Ampicillin | 7.000 | 5.595 to 8.405 | Yes | **** | <0.0001 |  |  |  |
| A. dubius vs. A. blitoides var. blitoides | -5.333 | -6.739 to -3.928 | Yes | **** | <0.0001 |  |  |  |
| A. dubius vs. A. viridis | -0.6667 | -2.072 to 0.7386 | No | ns | 0.3460 |  |  |  |
| A. dubius vs. Silver nitrate | 11.00 | 9.595 to 12.41 | Yes | **** | <0.0001 |  |  |  |
| A. dubius vs. Ampicillin | 6.667 | 5.261 to 8.072 | Yes | **** | <0.0001 |  |  |  |
| A. blitoides var. blitoides vs. A. viridis | 4.667 | 3.261 to 6.072 | Yes | **** | <0.0001 |  |  |  |
| A. blitoides var. blitoides vs. Silver nitrate | 16.33 | 14.93 to 17.74 | Yes | **** | <0.0001 |  |  |  |
| A. blitoides var. blitoides vs. Ampicillin | 12.00 | 10.59 to 13.41 | Yes | **** | <0.0001 |  |  |  |
| A. viridis vs. Silver nitrate | 11.67 | 10.26 to 13.07 | Yes | **** | <0.0001 |  |  |  |
| A. viridis vs. Ampicillin | 7.333 | 5.928 to 8.739 | Yes | **** | <0.0001 |  |  |  |
| Silver nitrate vs. Ampicillin | -4.333 | -5.739 to -2.928 | Yes | **** | <0.0001 |  |  |  |
|  |  |  |  |  |  |  |  |  |
| P. aeruginosa |  |  |  |  |  |  |  |  |
| Group A vs. Group B | -5.000 | -6.405 to -3.595 | Yes | **** | <0.0001 |  |  |  |
| Group A vs. A. dubius | -8.667 | -10.07 to -7.261 | Yes | **** | <0.0001 |  |  |  |
| Group A vs. A. blitoides var. blitoides | -7.667 | -9.072 to -6.261 | Yes | **** | <0.0001 |  |  |  |
| Group A vs. A. viridis | -9.667 | -11.07 to -8.261 | Yes | **** | <0.0001 |  |  |  |
| Group A vs. Silver nitrate | 9.000 | 7.595 to 10.41 | Yes | **** | <0.0001 |  |  |  |
| Group A vs. Ampicillin | 6.333 | 4.928 to 7.739 | Yes | **** | <0.0001 |  |  |  |
| Group B vs. A. dubius | -3.667 | -5.072 to -2.261 | Yes | **** | <0.0001 |  |  |  |
| Group B vs. A. blitoides var. blitoides | -2.667 | -4.072 to -1.261 | Yes | *** | 0.0004 |  |  |  |
| Group B vs. A. viridis | -4.667 | -6.072 to -3.261 | Yes | **** | <0.0001 |  |  |  |
| Group B vs. Silver nitrate | 14.00 | 12.59 to 15.41 | Yes | **** | <0.0001 |  |  |  |
| Group B vs. Ampicillin | 11.33 | 9.928 to 12.74 | Yes | **** | <0.0001 |  |  |  |
| A. dubius vs. A. blitoides var. blitoides | 1.000 | -0.4052 to 2.405 | No | ns | 0.1595 |  |  |  |
| A. dubius vs. A. viridis | -1.000 | -2.405 to 0.4052 | No | ns | 0.1595 |  |  |  |
| A. dubius vs. Silver nitrate | 17.67 | 16.26 to 19.07 | Yes | **** | <0.0001 |  |  |  |
| A. dubius vs. Ampicillin | 15.00 | 13.59 to 16.41 | Yes | **** | <0.0001 |  |  |  |
| A. blitoides var. blitoides vs. A. viridis | -2.000 | -3.405 to -0.5948 | Yes | ** | 0.0061 |  |  |  |
| A. blitoides var. blitoides vs. Silver nitrate | 16.67 | 15.26 to 18.07 | Yes | **** | <0.0001 |  |  |  |
| A. blitoides var. blitoides vs. Ampicillin | 14.00 | 12.59 to 15.41 | Yes | **** | <0.0001 |  |  |  |
| A. viridis vs. Silver nitrate | 18.67 | 17.26 to 20.07 | Yes | **** | <0.0001 |  |  |  |
| A. viridis vs. Ampicillin | 16.00 | 14.59 to 17.41 | Yes | **** | <0.0001 |  |  |  |
| Silver nitrate vs. Ampicillin | -2.667 | -4.072 to -1.261 | Yes | *** | 0.0004 |  |  |  |
|  |  |  |  |  |  |  |  |  |
| E. coli |  |  |  |  |  |  |  |  |
| Group A vs. Group B | 0.000 | -1.405 to 1.405 | No | ns | >0.9999 |  |  |  |
| Group A vs. A. dubius | -6.000 | -7.405 to -4.595 | Yes | **** | <0.0001 |  |  |  |
| Group A vs. A. blitoides var. blitoides | -17.00 | -18.41 to -15.59 | Yes | **** | <0.0001 |  |  |  |
| Group A vs. A. viridis | -8.000 | -9.405 to -6.595 | Yes | **** | <0.0001 |  |  |  |
| Group A vs. Silver nitrate | 11.67 | 10.26 to 13.07 | Yes | **** | <0.0001 |  |  |  |
| Group A vs. Ampicillin | 7.000 | 5.595 to 8.405 | Yes | **** | <0.0001 |  |  |  |
| Group B vs. A. dubius | -6.000 | -7.405 to -4.595 | Yes | **** | <0.0001 |  |  |  |
| Group B vs. A. blitoides var. blitoides | -17.00 | -18.41 to -15.59 | Yes | **** | <0.0001 |  |  |  |
| Group B vs. A. viridis | -8.000 | -9.405 to -6.595 | Yes | **** | <0.0001 |  |  |  |
| Group B vs. Silver nitrate | 11.67 | 10.26 to 13.07 | Yes | **** | <0.0001 |  |  |  |
| Group B vs. Ampicillin | 7.000 | 5.595 to 8.405 | Yes | **** | <0.0001 |  |  |  |
| A. dubius vs. A. blitoides var. blitoides | -11.00 | -12.41 to -9.595 | Yes | **** | <0.0001 |  |  |  |
| A. dubius vs. A. viridis | -2.000 | -3.405 to -0.5948 | Yes | ** | 0.0061 |  |  |  |
| A. dubius vs. Silver nitrate | 17.67 | 16.26 to 19.07 | Yes | **** | <0.0001 |  |  |  |
| A. dubius vs. Ampicillin | 13.00 | 11.59 to 14.41 | Yes | **** | <0.0001 |  |  |  |
| A. blitoides var. blitoides vs. A. viridis | 9.000 | 7.595 to 10.41 | Yes | **** | <0.0001 |  |  |  |
| A. blitoides var. blitoides vs. Silver nitrate | 28.67 | 27.26 to 30.07 | Yes | **** | <0.0001 |  |  |  |
| A. blitoides var. blitoides vs. Ampicillin | 24.00 | 22.59 to 25.41 | Yes | **** | <0.0001 |  |  |  |
| A. viridis vs. Silver nitrate | 19.67 | 18.26 to 21.07 | Yes | **** | <0.0001 |  |  |  |
| A. viridis vs. Ampicillin | 15.00 | 13.59 to 16.41 | Yes | **** | <0.0001 |  |  |  |
| Silver nitrate vs. Ampicillin | -4.667 | -6.072 to -3.261 | Yes | **** | <0.0001 |  |  |  |
|  |  |  |  |  |  |  |  |  |
|  |  |  |  |  |  |  |  |  |
| Test details | Mean 1 | Mean 2 | Mean Diff. | SE of diff. | N1 | N2 | t | DF |
|  |  |  |  |  |  |  |  |  |
| S. aureus |  |  |  |  |  |  |  |  |
| Group A vs. Group B | 16.00 | 18.00 | -2.000 | 0.7015 | 3 | 3 | 2.851 | 56.00 |
| Group A vs. A. dubius | 16.00 | 27.00 | -11.00 | 0.7015 | 3 | 3 | 15.68 | 56.00 |
| Group A vs. A. blitoides var. blitoides | 16.00 | 24.00 | -8.000 | 0.7015 | 3 | 3 | 11.40 | 56.00 |
| Group A vs. A. viridis | 16.00 | 26.00 | -10.00 | 0.7015 | 3 | 3 | 14.26 | 56.00 |
| Group A vs. Silver nitrate | 16.00 | 10.33 | 5.667 | 0.7015 | 3 | 3 | 8.078 | 56.00 |
| Group A vs. Ampicillin | 16.00 | 12.00 | 4.000 | 0.7015 | 3 | 3 | 5.702 | 56.00 |
| Group B vs. A. dubius | 18.00 | 27.00 | -9.000 | 0.7015 | 3 | 3 | 12.83 | 56.00 |
| Group B vs. A. blitoides var. blitoides | 18.00 | 24.00 | -6.000 | 0.7015 | 3 | 3 | 8.553 | 56.00 |
| Group B vs. A. viridis | 18.00 | 26.00 | -8.000 | 0.7015 | 3 | 3 | 11.40 | 56.00 |
| Group B vs. Silver nitrate | 18.00 | 10.33 | 7.667 | 0.7015 | 3 | 3 | 10.93 | 56.00 |
| Group B vs. Ampicillin | 18.00 | 12.00 | 6.000 | 0.7015 | 3 | 3 | 8.553 | 56.00 |
| A. dubius vs. A. blitoides var. blitoides | 27.00 | 24.00 | 3.000 | 0.7015 | 3 | 3 | 4.277 | 56.00 |
| A. dubius vs. A. viridis | 27.00 | 26.00 | 1.000 | 0.7015 | 3 | 3 | 1.426 | 56.00 |
| A. dubius vs. Silver nitrate | 27.00 | 10.33 | 16.67 | 0.7015 | 3 | 3 | 23.76 | 56.00 |
| A. dubius vs. Ampicillin | 27.00 | 12.00 | 15.00 | 0.7015 | 3 | 3 | 21.38 | 56.00 |
| A. blitoides var. blitoides vs. A. viridis | 24.00 | 26.00 | -2.000 | 0.7015 | 3 | 3 | 2.851 | 56.00 |
| A. blitoides var. blitoides vs. Silver nitrate | 24.00 | 10.33 | 13.67 | 0.7015 | 3 | 3 | 19.48 | 56.00 |
| A. blitoides var. blitoides vs. Ampicillin | 24.00 | 12.00 | 12.00 | 0.7015 | 3 | 3 | 17.11 | 56.00 |
| A. viridis vs. Silver nitrate | 26.00 | 10.33 | 15.67 | 0.7015 | 3 | 3 | 22.33 | 56.00 |
| A. viridis vs. Ampicillin | 26.00 | 12.00 | 14.00 | 0.7015 | 3 | 3 | 19.96 | 56.00 |
| Silver nitrate vs. Ampicillin | 10.33 | 12.00 | -1.667 | 0.7015 | 3 | 3 | 2.376 | 56.00 |
|  |  |  |  |  |  |  |  |  |
| k. pneumoniae |  |  |  |  |  |  |  |  |
| Group A vs. Group B | 18.00 | 20.00 | -2.000 | 0.7015 | 3 | 3 | 2.851 | 56.00 |
| Group A vs. A. dubius | 18.00 | 19.67 | -1.667 | 0.7015 | 3 | 3 | 2.376 | 56.00 |
| Group A vs. A. blitoides var. blitoides | 18.00 | 25.00 | -7.000 | 0.7015 | 3 | 3 | 9.979 | 56.00 |
| Group A vs. A. viridis | 18.00 | 20.33 | -2.333 | 0.7015 | 3 | 3 | 3.326 | 56.00 |
| Group A vs. Silver nitrate | 18.00 | 8.667 | 9.333 | 0.7015 | 3 | 3 | 13.31 | 56.00 |
| Group A vs. Ampicillin | 18.00 | 13.00 | 5.000 | 0.7015 | 3 | 3 | 7.128 | 56.00 |
| Group B vs. A. dubius | 20.00 | 19.67 | 0.3333 | 0.7015 | 3 | 3 | 0.4752 | 56.00 |
| Group B vs. A. blitoides var. blitoides | 20.00 | 25.00 | -5.000 | 0.7015 | 3 | 3 | 7.128 | 56.00 |
| Group B vs. A. viridis | 20.00 | 20.33 | -0.3333 | 0.7015 | 3 | 3 | 0.4752 | 56.00 |
| Group B vs. Silver nitrate | 20.00 | 8.667 | 11.33 | 0.7015 | 3 | 3 | 16.16 | 56.00 |
| Group B vs. Ampicillin | 20.00 | 13.00 | 7.000 | 0.7015 | 3 | 3 | 9.979 | 56.00 |
| A. dubius vs. A. blitoides var. blitoides | 19.67 | 25.00 | -5.333 | 0.7015 | 3 | 3 | 7.603 | 56.00 |
| A. dubius vs. A. viridis | 19.67 | 20.33 | -0.6667 | 0.7015 | 3 | 3 | 0.9504 | 56.00 |
| A. dubius vs. Silver nitrate | 19.67 | 8.667 | 11.00 | 0.7015 | 3 | 3 | 15.68 | 56.00 |
| A. dubius vs. Ampicillin | 19.67 | 13.00 | 6.667 | 0.7015 | 3 | 3 | 9.504 | 56.00 |
| A. blitoides var. blitoides vs. A. viridis | 25.00 | 20.33 | 4.667 | 0.7015 | 3 | 3 | 6.653 | 56.00 |
| A. blitoides var. blitoides vs. Silver nitrate | 25.00 | 8.667 | 16.33 | 0.7015 | 3 | 3 | 23.28 | 56.00 |
| A. blitoides var. blitoides vs. Ampicillin | 25.00 | 13.00 | 12.00 | 0.7015 | 3 | 3 | 17.11 | 56.00 |
| A. viridis vs. Silver nitrate | 20.33 | 8.667 | 11.67 | 0.7015 | 3 | 3 | 16.63 | 56.00 |
| A. viridis vs. Ampicillin | 20.33 | 13.00 | 7.333 | 0.7015 | 3 | 3 | 10.45 | 56.00 |
| Silver nitrate vs. Ampicillin | 8.667 | 13.00 | -4.333 | 0.7015 | 3 | 3 | 6.177 | 56.00 |
|  |  |  |  |  |  |  |  |  |
| P. aeruginosa |  |  |  |  |  |  |  |  |
| Group A vs. Group B | 17.33 | 22.33 | -5.000 | 0.7015 | 3 | 3 | 7.128 | 56.00 |
| Group A vs. A. dubius | 17.33 | 26.00 | -8.667 | 0.7015 | 3 | 3 | 12.35 | 56.00 |
| Group A vs. A. blitoides var. blitoides | 17.33 | 25.00 | -7.667 | 0.7015 | 3 | 3 | 10.93 | 56.00 |
| Group A vs. A. viridis | 17.33 | 27.00 | -9.667 | 0.7015 | 3 | 3 | 13.78 | 56.00 |
| Group A vs. Silver nitrate | 17.33 | 8.333 | 9.000 | 0.7015 | 3 | 3 | 12.83 | 56.00 |
| Group A vs. Ampicillin | 17.33 | 11.00 | 6.333 | 0.7015 | 3 | 3 | 9.029 | 56.00 |
| Group B vs. A. dubius | 22.33 | 26.00 | -3.667 | 0.7015 | 3 | 3 | 5.227 | 56.00 |
| Group B vs. A. blitoides var. blitoides | 22.33 | 25.00 | -2.667 | 0.7015 | 3 | 3 | 3.802 | 56.00 |
| Group B vs. A. viridis | 22.33 | 27.00 | -4.667 | 0.7015 | 3 | 3 | 6.653 | 56.00 |
| Group B vs. Silver nitrate | 22.33 | 8.333 | 14.00 | 0.7015 | 3 | 3 | 19.96 | 56.00 |
| Group B vs. Ampicillin | 22.33 | 11.00 | 11.33 | 0.7015 | 3 | 3 | 16.16 | 56.00 |
| A. dubius vs. A. blitoides var. blitoides | 26.00 | 25.00 | 1.000 | 0.7015 | 3 | 3 | 1.426 | 56.00 |
| A. dubius vs. A. viridis | 26.00 | 27.00 | -1.000 | 0.7015 | 3 | 3 | 1.426 | 56.00 |
| A. dubius vs. Silver nitrate | 26.00 | 8.333 | 17.67 | 0.7015 | 3 | 3 | 25.19 | 56.00 |
| A. dubius vs. Ampicillin | 26.00 | 11.00 | 15.00 | 0.7015 | 3 | 3 | 21.38 | 56.00 |
| A. blitoides var. blitoides vs. A. viridis | 25.00 | 27.00 | -2.000 | 0.7015 | 3 | 3 | 2.851 | 56.00 |
| A. blitoides var. blitoides vs. Silver nitrate | 25.00 | 8.333 | 16.67 | 0.7015 | 3 | 3 | 23.76 | 56.00 |
| A. blitoides var. blitoides vs. Ampicillin | 25.00 | 11.00 | 14.00 | 0.7015 | 3 | 3 | 19.96 | 56.00 |
| A. viridis vs. Silver nitrate | 27.00 | 8.333 | 18.67 | 0.7015 | 3 | 3 | 26.61 | 56.00 |
| A. viridis vs. Ampicillin | 27.00 | 11.00 | 16.00 | 0.7015 | 3 | 3 | 22.81 | 56.00 |
| Silver nitrate vs. Ampicillin | 8.333 | 11.00 | -2.667 | 0.7015 | 3 | 3 | 3.802 | 56.00 |
|  |  |  |  |  |  |  |  |  |
| E. coli |  |  |  |  |  |  |  |  |
| Group A vs. Group B | 20.00 | 20.00 | 0.000 | 0.7015 | 3 | 3 | 0.000 | 56.00 |
| Group A vs. A. dubius | 20.00 | 26.00 | -6.000 | 0.7015 | 3 | 3 | 8.553 | 56.00 |
| Group A vs. A. blitoides var. blitoides | 20.00 | 37.00 | -17.00 | 0.7015 | 3 | 3 | 24.23 | 56.00 |
| Group A vs. A. viridis | 20.00 | 28.00 | -8.000 | 0.7015 | 3 | 3 | 11.40 | 56.00 |
| Group A vs. Silver nitrate | 20.00 | 8.333 | 11.67 | 0.7015 | 3 | 3 | 16.63 | 56.00 |
| Group A vs. Ampicillin | 20.00 | 13.00 | 7.000 | 0.7015 | 3 | 3 | 9.979 | 56.00 |
| Group B vs. A. dubius | 20.00 | 26.00 | -6.000 | 0.7015 | 3 | 3 | 8.553 | 56.00 |
| Group B vs. A. blitoides var. blitoides | 20.00 | 37.00 | -17.00 | 0.7015 | 3 | 3 | 24.23 | 56.00 |
| Group B vs. A. viridis | 20.00 | 28.00 | -8.000 | 0.7015 | 3 | 3 | 11.40 | 56.00 |
| Group B vs. Silver nitrate | 20.00 | 8.333 | 11.67 | 0.7015 | 3 | 3 | 16.63 | 56.00 |
| Group B vs. Ampicillin | 20.00 | 13.00 | 7.000 | 0.7015 | 3 | 3 | 9.979 | 56.00 |
| A. dubius vs. A. blitoides var. blitoides | 26.00 | 37.00 | -11.00 | 0.7015 | 3 | 3 | 15.68 | 56.00 |
| A. dubius vs. A. viridis | 26.00 | 28.00 | -2.000 | 0.7015 | 3 | 3 | 2.851 | 56.00 |
| A. dubius vs. Silver nitrate | 26.00 | 8.333 | 17.67 | 0.7015 | 3 | 3 | 25.19 | 56.00 |
| A. dubius vs. Ampicillin | 26.00 | 13.00 | 13.00 | 0.7015 | 3 | 3 | 18.53 | 56.00 |
| A. blitoides var. blitoides vs. A. viridis | 37.00 | 28.00 | 9.000 | 0.7015 | 3 | 3 | 12.83 | 56.00 |
| A. blitoides var. blitoides vs. Silver nitrate | 37.00 | 8.333 | 28.67 | 0.7015 | 3 | 3 | 40.87 | 56.00 |
| A. blitoides var. blitoides vs. Ampicillin | 37.00 | 13.00 | 24.00 | 0.7015 | 3 | 3 | 34.21 | 56.00 |
| A. viridis vs. Silver nitrate | 28.00 | 8.333 | 19.67 | 0.7015 | 3 | 3 | 28.04 | 56.00 |
| A. viridis vs. Ampicillin | 28.00 | 13.00 | 15.00 | 0.7015 | 3 | 3 | 21.38 | 56.00 |
| Silver nitrate vs. Ampicillin | 8.333 | 13.00 | -4.667 | 0.7015 | 3 | 3 | 6.653 | 56.00 |
